# Supplementary figures and images for: Scalable Hypothalamic Arcuate Neuron Differentiation from Human Pluripotent Stem Cells Suitable for Modeling Metabolic and Reproductive Disorders
Source: bioRxiv. 2024 Sep 20:2024.06.27.601062. Originally published 2024 Jul 2. Preprint. [Version 2] doi: 10.1101/2024.06.27.601062 (PMC11244856; doi:10.1101/2024.06.27.601062)

Figure S1

A

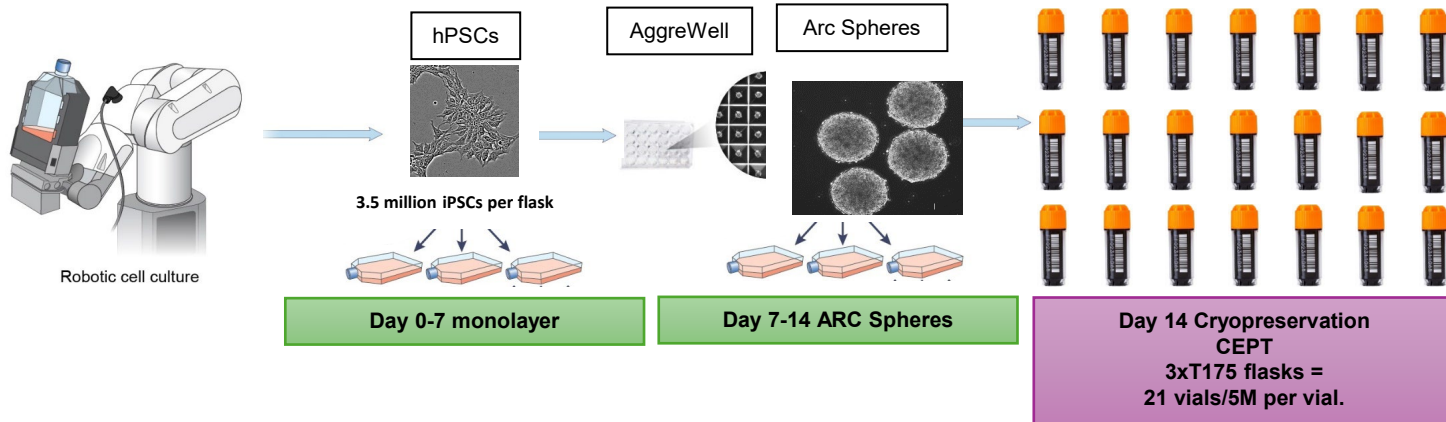

B

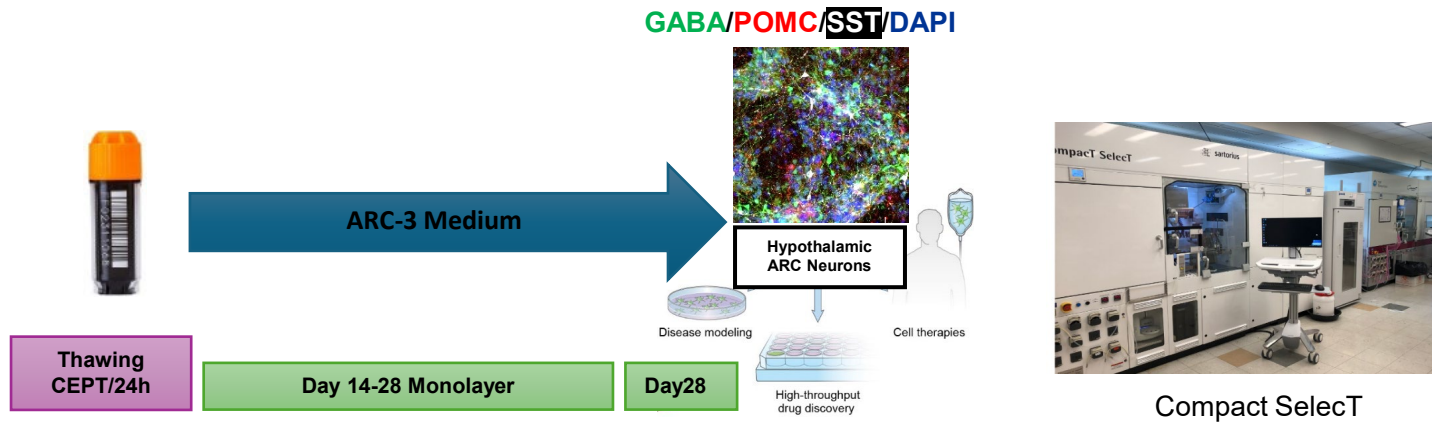

Figure S2

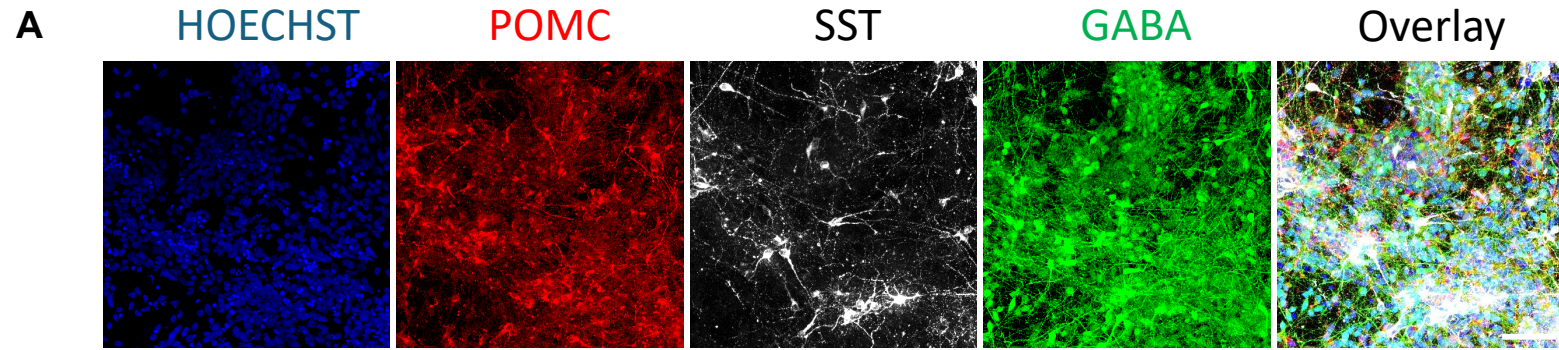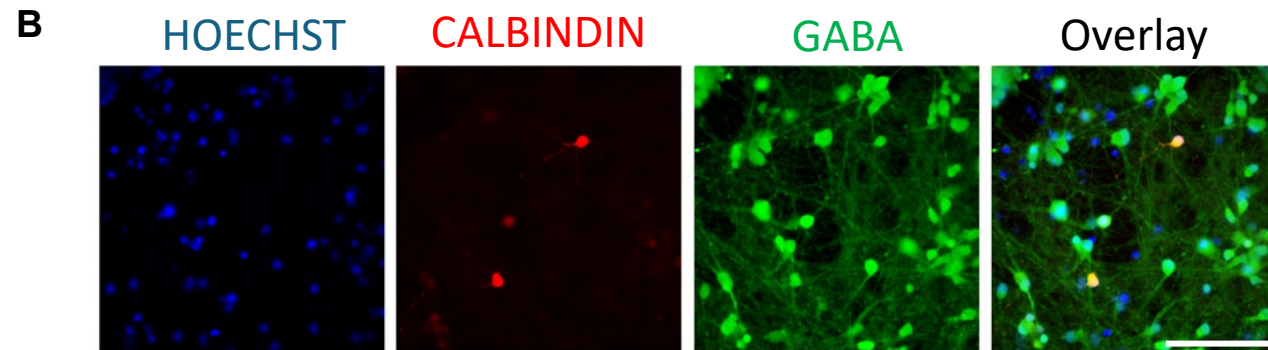

Figure S3

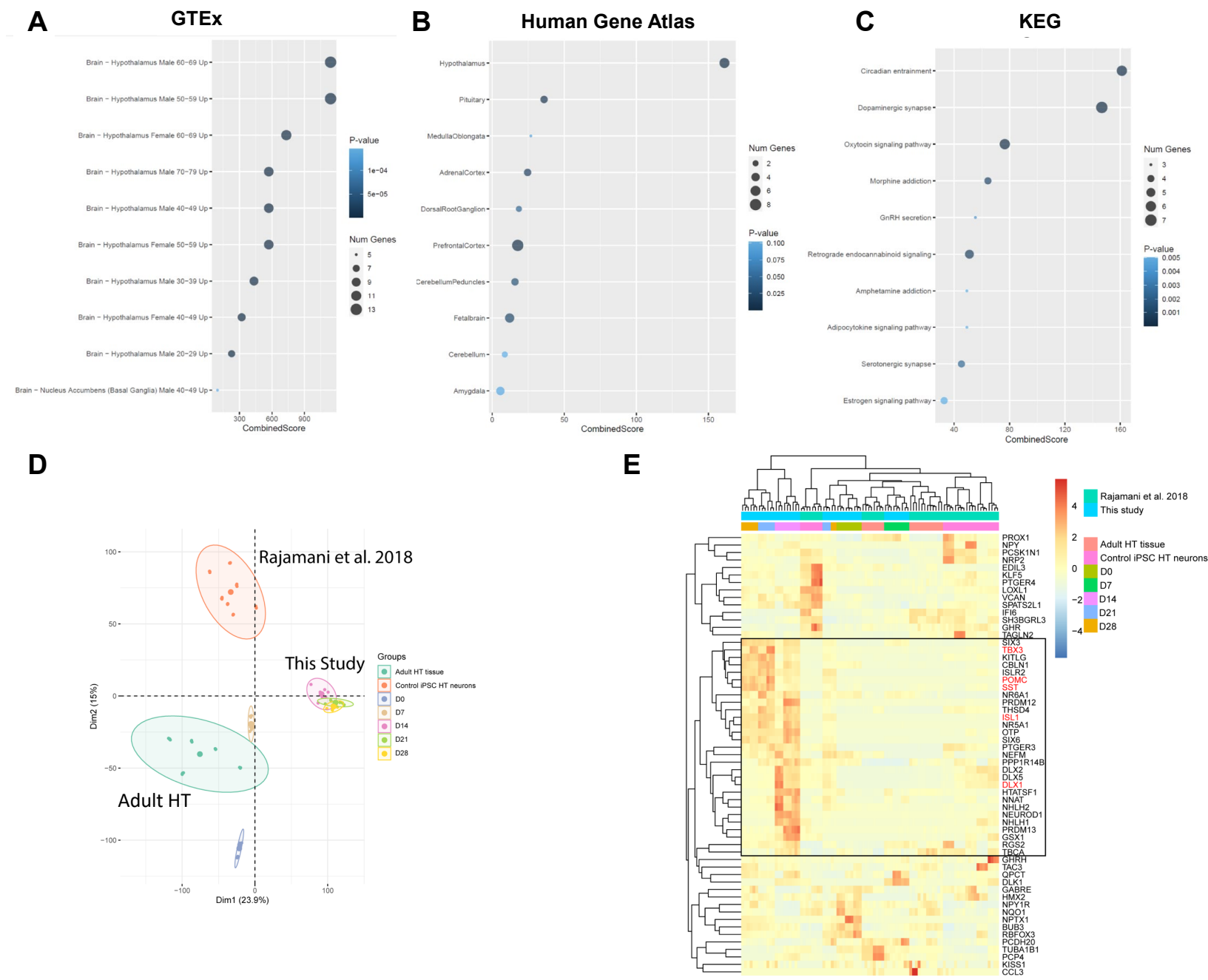

Figure S4

**A**

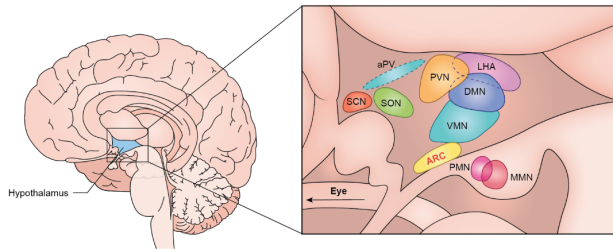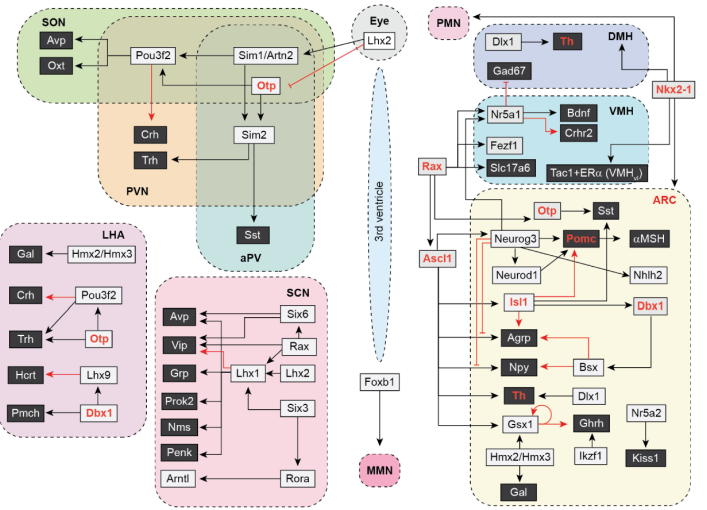

**B**

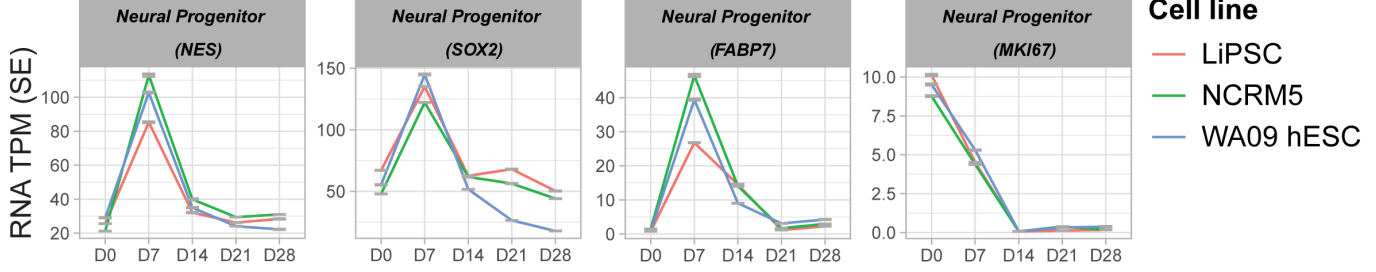

**C**

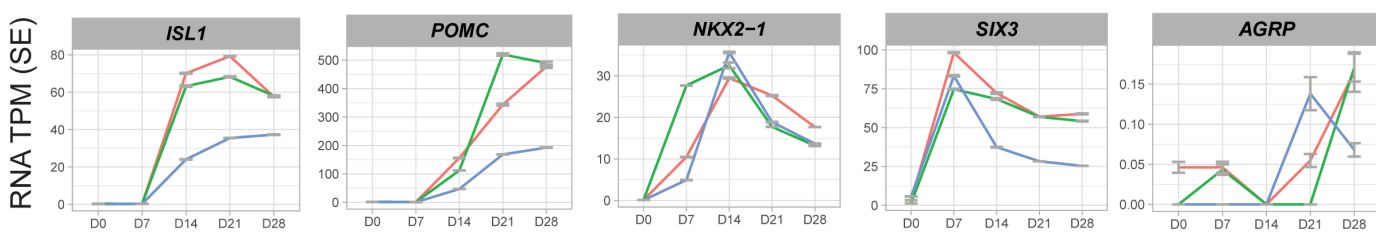

**D**

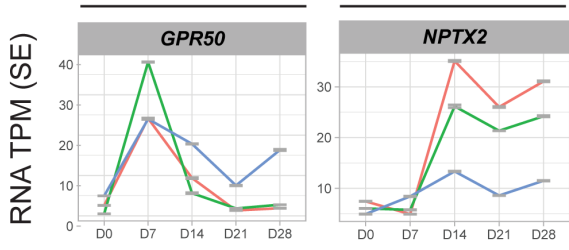

**E**

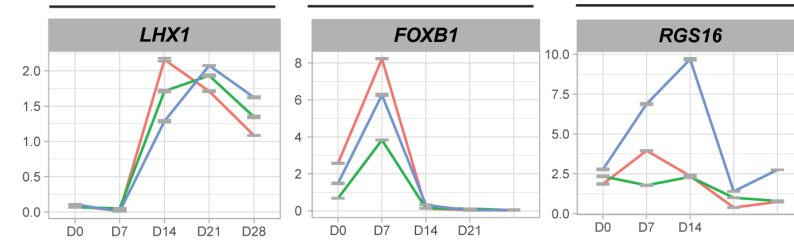

**F**

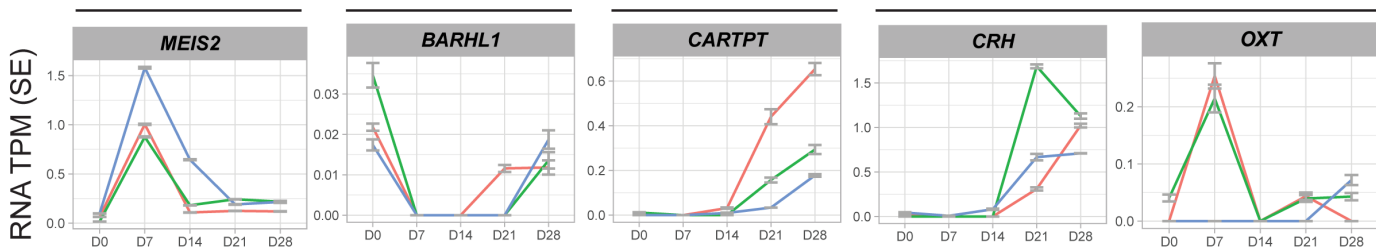

# Figure S5

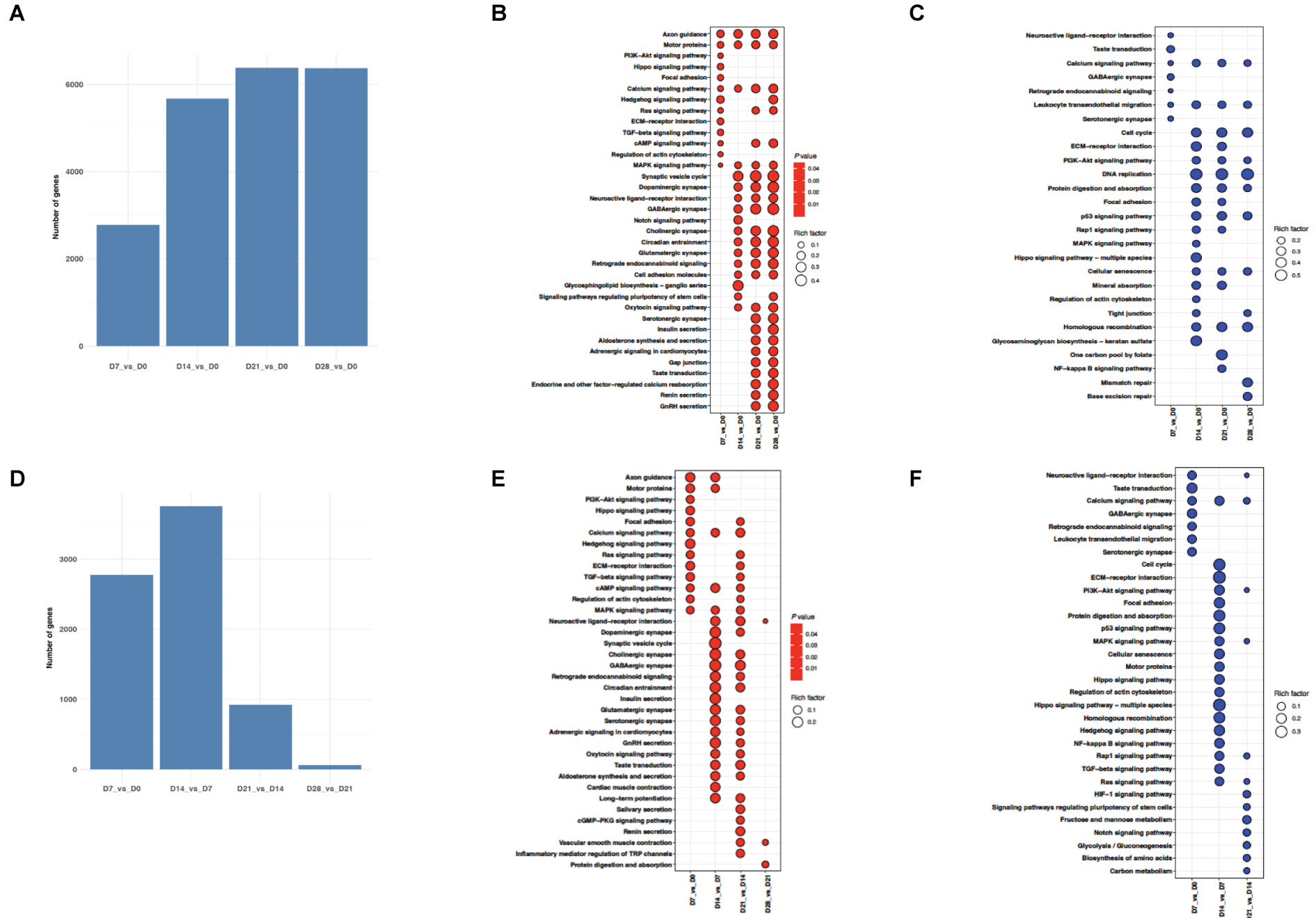

Supplement: Supplement 2 [file media-2.pdf]
